# Effect of Nanoclay on Thermal Conductivity and Flexural Strength of Polymethyl Methacrylate Acrylic Resin

**Published:** 2016-06

**Authors:** Tahereh Ghaffari, Ali Barzegar, Fahimeh Hamedi Rad, Elnaz Moslehifard

**Affiliations:** 1 Periodontal Research Center, Dept. of Prosthodontics, Tabriz University of Medical Sciences, Tabriz, Iran.; 2 Dept. of Prosthodontics, Faculty of Dentistry, Qom University of Medical Sciences, Qom, Iran.

**Keywords:** Polymethyl Methacrylate, Nanoparticles, Thermal Conductivity, Flexural Strength

## Abstract

**Statement of the Problem:**

The mechanical and thermal properties of polymethyl methacrylate (PMMA) acrylic resin should be improved to counterweigh its structural deficiencies.

**Purpose:**

The aim of this study was to compare the flexural strength and thermal conductivity of conventional acrylic resin and acrylic resin loaded with nanoclay.

**Materials and Method:**

The methacrylate monomer containing the 0.5, 1 and 2 wt% of nanoclay was placed in an ultrasonic probe and mixed with the PMMA powder. Scanning electron microscopy was used to verify homogeneous distribution of particles. Twenty-four 20×20×200-mm cubic samples were prepared for flexural strength test; 18 samples containing nanoclay and 6 samples for the control group. Another 24 cylindrical samples of 38×25 mm were prepared for thermal conductivity test. One-way ANOVA was used for statistical analysis, followed by multiple-comparison test (Scheffé’s test). Statistical significance was set at *p*< 0.05.

**Results:**

Increasing the concentration of nanoclay incorporated into the acrylic resin samples increased thermal conductivity but decreased flexural strength (*p*< 0.05).

**Conclusion:**

Based on the results of this study, adding nanoclay particles to PMMA improved its thermal conductivity, while it had a negative effect on the flexural strength.

## Introduction

In recent years, perceiving the advances in the nanotechnology science, many attempts have been made to use these particles in dental acrylic resin to improve its fatigue behavior, impact strength, and thermal conductivity, and some successes have been achieved.[1] However, there are some discrepancies between the results of these studies.[1-3]

Thermal conductivity is an important feature of acrylic resin which affects prosthesis acceptance by the patient. Besides, it has major impacts on salivary glands secretion (especially parotid gland) and health of denture supporting tissues. One of the main drawbacks of polymethyl methacrylate (PMMA) resin is its low thermal conductivity.[4-5]

Nano titanium dioxide can be incorporated into dental materials to mimic the opacity of teeth[1] or to decrease bacterial adhesion to these materials.[2] However, studies show that it cannot improve the mechanical properties of composite resins.[6-7] In addition, nano titanium dioxide can increase the hardness of acrylic resins used as temporary crowns.[3] Nanoclay is another material which is used to improve the properties of composite and acrylic polymers. Montmorillonite (MMT) is one of the available forms of nanoclay, which consists of small layers with internal octahedral layer interposed between two tetrasilicate layers. This structure is claimed to prevent formation of cracks and, therefore, it can improve flexural strength.[8]

Atai *et al.*[9] used PMMA-grafted nanoclay as filler for dental adhesives which increased the shear bond strength of the adhesive and improved the dispersion stability of the particles 40 folds compared to pure nanoclay.

De Polo & Baird[10] evaluated the effect of organoclay loading and the technique used for surface preparation of nanoparticles on the dimensional stability, flexural strength, and tensile toughness of different nano-composites. They reported that incorporation of organoclay with a polaramine composition at a concentration of 1% increased the flexural strength and tensile toughness by 12‒27%. However, improvements in flexural and tensile strengths of nano-composites occurred at higher concentrations of nanoparticles.

It was reported that incorporation of 0.5 wt% of nanoclay into the acrylic resin increased its yield strength and shear strength. Incorporation of higher concentrations of this material increased the shear modulus of the material.[8]

Mortazavi *et al.*[11] evaluated the effect of pure nanoclay fillers and PMMA-grafted nanoclay fillers on the flexural strength of (fiber reinforced composite) FRC resins. The results showed that the modified nanoclay fillers increased the flexural strength of FRC. However, incorporation of unmodified particles did not significantly affect the flexural strength and even at some concentrations, none of the two above-mentioned fillers altered the flexural strength.

Considering the results of these studies and given a lack of sufficient information about the effect of nanoclay particles on the mechanical properties and the thermal conductivity of acrylic resin denture bases, the present sturdy was conducted to evaluate the effect of addition of nanoclay at 0.5, 1 and 2 wt% concentrations on the flexural strength and thermal conductivity of acrylic resin. 

## Materials and Method

This research made use of polymethyl methacrylate (PMMA; SR Triplex Hot, Ivoclar Vivadent, Liechtenstein, Germany) as a heat-curing acrylic resin, and Cloisite 20A commercial nanoclay material with a diameter of <6 μm and a density of 1.77 g/mL (Southern clay Products Inc.; Austin, U.S.A) which was modified with quaternary ammonium salts. 

Nanoclay in three concentration groups at 0.5, 1 and 2 wt% were mixed with heat-curing acrylic resin. These concentrations were determined based on a pilot study which revealed that addition of less than 2% nanoclay to the acrylic resin increases thermal conductivity with no effect on its mechanical properties.

The sample size was determined according to the results of pilot study. A total of 48 samples, 24 for each test (flexural strength and thermal conductivity) were prepared. Samples of each test were divided into 4 groups (n=6). 

Group A with 6 specimens of pure acrylic resin as the control group, Group B with 6 specimens of PMMA mixed with 0.5wt% of nanoclay, Group C with 6 specimens of PMMA mixed with 1wt% of nanoclay, and  Group D with 6 specimens of PMMA mixed with 2 wt% of nanoclay were prepared. 

Before acrylic resin packing procedures, to achieve the best distribution quality, the monomer containing the specified wt% of nanoclay was placed in an ultrasonic probe (Hielscher Ultrasonics GmbH, UP200H, Germany) for 5 minutes;[12-13] and then mixed with the powder. After the paste achieved a doughy consistency, it was packed into steel molds. Then, the specimens were removed from the molds after being cured.

All the samples were polished with 400-grit emery paper (grades 320, 500, 800, Nippon Coated Abrasive; Aichi, Japan) to remove any excess acrylic resin.

Thermal conductivity measurement apparatus (Cussons thermal conductivity apparatus; UK) and universal flexural strength measurement apparatus (Gotech Inc.; Baton Rouge, LA, USA) were employed to determine the thermal conductivity and flexural strength of samples. Based on ASTM C177 (ISO 8302) standard recommended by the manufacturer of the measurement device, 24 thermal conductivity test samples were prepared. The specimens were formed in cylinders of 38×25 mm with a metal mold.[14-15] Another 24 specimens were prepared for flexural test in rectangular cubic shape, measuring 20×20×200mm according to ASTM D790-10 (ISO 178)  recommended by the manufacturer. All samples were measured by a digital caliper (Guanglu, Strikhlu, Germany) and error of ±0.03mm was considered insignificant.

**Figure 1:**
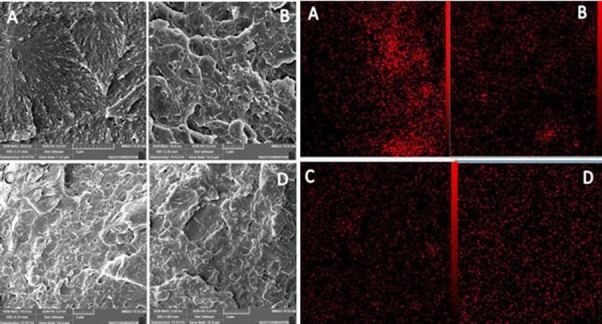
Scanning electron micrograph and mapping of samples a: Pure acrylic resin, b: 0.5% nanoclay,  c: 1% nanoclay,  d: 2% nanoclay

Scanning electron microscopy (SEM; VEGA/ TESCAN, Czech Republic) was used to study the distribution of nanoparticles and the cross-sectional morphology of the samples (Figure 1).

The specimens were conditioned in standard laboratory environment for 24 hours before performing the tests (temperature=23±2°C, humidity=50±5%).

To evaluate the thermal conductivity, two holes were produced, each 6.5 mm away from one end of the sample.[14-15] Then, the samples were placed in a heat conduction tester; two thermocouple systems were placed within these two holes. Water flew through holes and the difference in temperature between the two ends was determined by the thermometers of the tester. The thermal conduction coefficient (k) was calculated for each sample using following formula:

$$k=\;\frac{j\times M\times L\times (T_{2}-T_{1})}{A\times t\times (t_{2}-t_{3})}$$

where:

j= Mechanical equivalent of heat =0.186 j/Kcal

M= Water mass 

L= Length of specimen

A= Cross sectional area 

t= Time of water flow

T2= Temperature of output water

T1= Temperature of input water

t2= Temperature at the cold end

t3= Temperature at the hot end

Three-point flexural strength of samples were evaluated regarding the ASTM D790-10 criteria, with diameter of the pressure head of 3.5 mm, the sensor force of 10 kg, span of 170 mm, and loading speed of 2.4 mm/min. The extent of flexion of each sample was recorded in mm. Finally, the flexural strength was measured by using following formula:

$$\sigma =\frac{3FL}{{2db}^{2}}$$

where:

F = load (force) at the fracture point

L = length of the support span

b = width (MM)

d = thickness

The mean, average, and mode were calculated in each group and normal distribution curve was appraised. One-way ANOVA and then multiple-comparison test (Scheffé’s test) were used for statistical analysis. Statistical significance was set at *p*< 0.05.

## Results

The results of One-way ANOVA showed significant differences in thermal conduction values of the groups (Table 1). The results of multiple-comparison test for thermal conductivity showed that acrylic resin with 1% and 2% nanoclay concentrations(Group C) had a significantly higher thermal conductivity compared with the control group (*p*< 0.05). However, group B with 0.5% nanoclay concentration had a significantly lower thermal conductivity compared with other groups (*p*< 0.05).

**Table 1:** Means and standard deviations of thermal conduction values of the tested groups (in J/m.s.°C)

**Group**	**N**	**Mean**	**SD**	**Min**	**Max**	**ANOVA**	
						**F**	**P**
A (control)	6	4.3982	.02222	4.38	4.44	75.507	0.000
B (0.5%)	6	4.3380	.01585	4.32	4.36		
C (1%)	6	4.4590	.04273	4.41	4.54		
D (2%)	6	4.5603	.01697	4.54	4.58		

**Figure 2:**
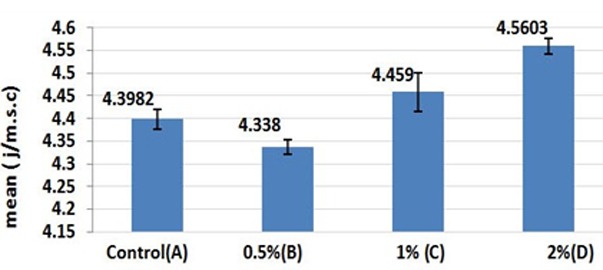
Comparison of thermal conductivity of specimens

The comparison of thermal conductivity results of all groups are illustrated in Figure 2.

**Figure 3:**
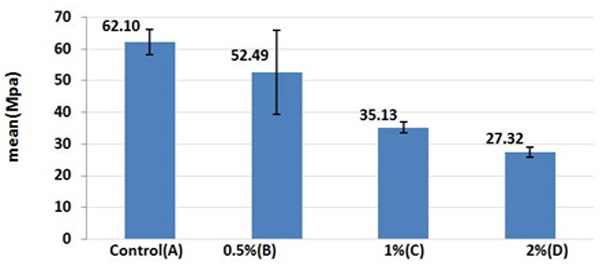
Comparison of flexural strengths of specimens

Table 2 presents the descriptive data of the flexural strength tests yielded from all samples in 4 groups. ANOVA showed significant differences in the mean flexural strength values among the 4 groups (*p*< 0.05). The results of multiple comparison test for flexural strength of samples in all groups showed that acrylic resin samples with 1% and 2% nanoclay concentrations (Group C) had a significantly lower flexural strength compared to the control group (*p*< 0.05). There were statistically significant differences in the mean flexural strength values of acrylic resin samples of Group B and Group D (*p*< 0.05). The flexural strength values of all groups are compared in Figure 3.

**Table 2:** Means and standard deviations of flexural strengths of the tested groups (in MPa)

**Group**	**N**	**Mean**	**SD**	**Min**	**Max**	**ANOVA**	
						**F**	**P**
A (control)	6	62.0983	12.98494	47.81	85.70	11.79	0.000
B (0.5%)	6	52.4883	13.21215	28.65	65.87		
C (1%)	6	35.1250	9.45496	16.99	41.82		
D (2%)	6	27.3217	8.97339	15.92	38.91		

**Scanning Electron Microscope Studies****

The morphology of samples in cross-section and mapping of samples with different contents of nanoclay are displayed in Figure 1. As shown, the samples of Group B exhibited more homogeneous dispersion compared to the samples in group C and D. These figures show that an increase in nanoclay caused aggregation of nanoparticles in the sample by extra content, resulting in changes in the fractured surface and the microcracks became visible at higher concentrations.

## Discussion

The addition of nanoclay particles statistically increased the thermal conductivity of specimens compared to the control group. On the other side, incorporation of nanoclay decreased the flexural strength in all groups that contained nanoclay; the obtained values were statistically significant different between the control group and the groups that contained 1 and 2 wt% of nanoclay.

An improvement in the thermal conductivity, especially in groups loaded with 1 and 2 wt% of nanoclay, might be attributed to the thermal conductivity property of nanoclay containing silicon (Si) and creation of pathways for thermal conduction by these particles within the acrylic resin.[8, 11, 16] Likewise, the significant decrease in thermal conductivity at 0.5% concentration might be attributed to inadequate amount of particles essential to alter the thermal conduction property.

Similar to this study, several studies reported an improvement in thermal conductivity by incorporating micro- and nano-fillers into the resin matrix.[17-19] The type of filler and its distribution within the resin matrix affect the thermal conductivity properties. Based on the results of a study reported by Brassel & Wischmann, incorporation of metallic particles including copper, silver and aluminum into acrylic resin increased the thermal conductivity differently which depended on the type of the metal used.[17] In a study by Ghaffari *et al.*, incorporation of silver nanoparticles into acrylic resin increased the thermal conductivity.[18] In addition, Hakamy *et al.* reported that loading with 1% concentration of nanoclay improved the thermal conductivity and flexural strength of FRC resin cement.[19]

In different studies,[20-21] it was proposed that the addition of fillers to resin matrix can improve the mechanical properties of resin-based materials. It should be emphasized that the type of the fillers, their physical orientation, and their adherence capacity to the resin phase affect the physical characteristics of resin-based materials.[22-23] Concerning the type of the filler, it can be suggested that hydrophilic nanoparticles cannot be appropriately dispersed and agglomerate in the resin phase. This phenomenon concentrates the stress on particular points in the resin phase and spreads the crack easily through the unfilled parts of matrix resin. This can be the main reason for crack propagation that leads to matrix fracture and reduction in flexural strength. These explanations clarify why nanoparticles did not cause significant changes in the flexural strength.

Based on Hernandez *et al.*'s study,[3] incorporation of titanium dioxide nano-filler to 4 types of acrylic polymer used for the fabrication of temporary restorations decreased the mechanical properties. In Solhi *et al.*'s[8] and Zukas *et al.*’[24] studies, results showed that by increasing the amount of nanoparticles to more than a particular point, the flexural strength faced a significant reduction. In addition, in a study by Mortazavi *et al.,*[11] a definite decrease in flexural modulus was observed in all the samples loaded with unmodified nanoclay except for the 0.2% group. In samples loaded with nanoparticles, an increase in filler concentration decreased the flexural modulus. These findings are in accordance with the results of the present study.

However, some studies reported an improvement in mechanical properties with an increase in the filler content of acrylic resins. Acosta *et al.*[25] showed that titanium oxide was an appropriate filler to improve the mechanical properties of acrylic resins. Solhi *et al.*[8] reported that incorporation of nanoclay filler reinforced with PMMA into adhesive resin improved its flexural modulus. They also observed that higher filler content increased the flexural modulus. A study by Chisholm *et al.*[26] showed that PMMA-grafted nanoclay filler loading could improve the flexural strength of FRCs. Moreover, in the study of Labella *et al.*, silane-treated hydroxyapatite powder was used with different filler contents and showed enhanced flexural strength, tensile strength and Vickers hardness.[27]

An important problem with the use of metal particles including nano-silver is a change in acrylic polymer color, which limits its use in the esthetic zone.[18, 28-29] However, the advantage of PMMA reinforced with nanoclay is the absence of color changes in the polymer at none of the concentrations, which makes its use possible in all the areas of prosthetic appliances. Another important advantage of this material is its low weight; all the incorporated samples had the same weigh as the control samples.

The appropriate separation and dispersion of filler particles in the resin matrix, especially in nanoscale, play an important role in the physical characteristics of resin-based materials. So, it is important to achieve homogeneous dispersion of nanoparticles within the matrix polymer because it affects the results. In the present study, electron microscope images and mapping of the failed surfaces were used to evaluate the distribution of nanoparticles in the samples. It was found that the agglomeration of particles increased with elevating the concentration of nanoparticles in the resin matrix. Nanoparticles have an inherent propensity for agglomeration and different techniques have been suggested to solve this inherent problem when being mixed with resin matrix,[8, 10, 18, 28] including the use of an amalgamator or the probe of an ultrasonic device. The latter was used in the present study and resulted in better separation of nanoparticles and distribution within the resin matrix. In the studies carried out by Ghaffari *et al.* and Shirkavand & Moslehifard, an amalgamator was used which was not able to properly disperse the nanoparticles.[18, 28] A decrease in mechanical properties might be related to surface preparation and modification of nanoparticles, which affect the hydrophilicity of these particles.

In addition, the type of nanoparticles affects the results of studies.[10] Nanoclay is available in different commercial forms. The Sodium Montmorillonite (Na-MMT) is an unmodified type which was used in a study conducted by Solhi *et al.*[8] Other modified commercial forms are 15A, 20A, 25A and 30B, which have  different effects on the mechanical and thermal properties.

In the present study, a decrease in mechanical properties at higher concentrations might be attributed to the quality of dispersion of nanoparticles with different surface characteristics within the resin matrix. Undoubtedly, unmodified nanoparticles or particles modified with polar or non-polar amine derivatives exhibit different effects within the resin matrix with an increase in concentration. 

Given the limitations of the present study, further studies that would investigate other forms of nanoclay or other concentrations are suggested. Moreover, other techniques for surface preparation of nanoparticles instead of mechanical techniques should also be investigated to improve dispersion within the acrylic resin. 

## Conclusion

In the present study, loading of acrylic resin with modified nanoclay at 1 and 2 wt% concentrations increased the thermal conductivity; however, it significantly decreased the flexural strength of the acrylic resin. 
